# Contrast sensitivity perimetry data from adults free of eye disease

**DOI:** 10.1016/j.dib.2016.06.012

**Published:** 2016-06-21

**Authors:** William H. Swanson, Mitchell W. Dul, Douglas G. Horner, Victor E. Malinovsky

**Affiliations:** Indiana University School of Optometry, SUNY College of Optometry, USA

**Keywords:** Perimetry, Contrast sensitivity, Visual field

## Abstract

This data article contains data referenced in “Individual Differences in the Shape of the Nasal Visual Field” [1]. The data were gathered from volunteers free of eye disease ages 21–85 who were tested with Contrast Sensitivity Perimetry (CSP), which uses a stimulus resistant to effects of defocus and reduced retinal illumination. Some subjects were tested only once or a few times, and others were part of a longitudinal cohort with as many as 10 tests. Parameters from maximum likelihood estimation of psychophysical threshold at each tested location are included in the data file, along with the participant׳s sex, age at time of test, the center of their physiological blind spot, the duration of test, the time of day that the test was begun, and the starting contrast used for the psychophysical staircases.

**Specifications Table**

**Table:** 

Subject area	*Vision Science*
More specific subject area	*Perimetry*
Type of data	*Figure, excel File*
How data were acquired	*Perimetric testing on custom testing stations*
Data format	*Raw, filtered, and analyzed*
Experimental factors	*A four-reversal staircase was used to gather data, and maximum likelihood estimation was used to define the psychometric function in terms of threshold, slope, and upper asymptote*
Experimental features	*Volunteers tested for psychophysical contrast sensitivity using a stimulus resistant to effects of peripheral defocus and reduced illumination*
Data source location	*Bloomington, Indiana, USA; Indianapolis, Indiana, USA; New York City, New York, USA*
Data accessibility	*Dataset is with this article*

**Value of the data**

• The stimulus was engineered to be resistant to effects of peripheral defocus and reduced retinal illumination, and effects of age were less than for conventional perimetry, so these data may help differentiate effects of aging on visual optics from neural effects.
• These data indicate that studies of damage to the temporal raphe of the retinal nerve fiber layer may encounter challenges when referencing to mean normal sensitivity at individual locations rather than individual variability in shape of the visual field.
• These data could be helpful for developing improved methods estimating the effects of a person׳s decision criteria (variation in height of the hill of vision), leading to an improved Pattern Deviation (PD) index for perimetry.

## 1 Data

Contrast sensitivities at 26 locations in the central visual field, with an emphasis on the nasal field, for 107 people tested prospectively over periods ranging from 0.0 to 2.8 years, with 43% tested over at least 1 year and 29% tested over at least 2 years. [1]. For each test, the data include: sex, age, spherical lens used for test distance, false positive rate, false negative rate, fixation losses, slope of psychometric function, mean of reversals, difference between mean of reversals and 50% seen contrast for the psychometric function. [2].

## 2 Experimental design

Prospective, longitudinal data collection of functional and structural measures in patients with glaucoma, as well as age-similar controls followed with the same protocol as for the patients. This was part of NIH grant R01EY007716, and the goal for patients and age-similar controls was two CSP tests ~1 week apart (for test-retest reliability) every ~6 months for 1–2 years. During data collection the grant was transferred from SUNY College of Optometry to Indiana University (IU) School of Optometry, with a subcontract back to SUNY to continue following subjects. This meant that subjects at SUNY were tested until funding ended for the subcontract, while new subjects were recruited at IU and followed with additional funding from IU. When an improved method was developed [3], additional data with CSP were gathered to validate the new method on patients and controls who were not followed longitudinally.

CSP data were gathered from younger controls (21–30 years of age) in order to develop the CSP method [4] and to quantify the effects of optical defocus [5] and reduced retinal illumination. [6]. These data are from those CSP tests that were for the control condition (no blur or reduction in illumination). The age range for controls was 21–85, and the number of CSP tests varied from 1 to 11; half had at least 3 tests and a quarter had at least 6 tests.

## 3 Materials and methods

### 3.1 Participants

Over the duration of the multi-center data collection, volunteers were tested at four different locations. Three locations were at Indiana University School of Optometry and one location was at the State University of New York (SUNY) College of Optometry. The research for this data collection adhered to the tenets of the Declaration of Helsinki and was approved by the institutional review boards at Indiana University and SUNY College of Optometry. Informed consent was obtained from each participant after explanation of the procedures and goals of the data collection, before testing began.

Volunteers were recruited in the age range 21–85 years and were required to have regular eye exams, be free of visual disorder, have spherical equivalent refractive error between −6 D and +2 D, with cylinder <=−2.5 D, and corrected visual acuity of 20/20 or better (20/25 over age 70). These volunteers were experienced and reliable on perimetric testing. [3], [7].

Volunteers were tested with contrast sensitivity perimetry (CSP), which refers to perimetry with Gabor stimuli [8]. The 107 volunteers ranged in age from 21 to 86 yrs (median 60 yrs) and participated in testing from 1 to 10 times, over periods ranging from 0.0 to 2.8 years.

### 3.2 Equipment

Custom testing stations were used during the longitudinal investigation. The details of stimulus display, calibration, fixation monitoring, refractive correction for ametropia and test distance, stimulus configuration, test protocol and threshold algorithm for the CSP testing are available elsewhere [3], [4], [5].

### 3.3 Stimuli

The CSP stimulus [4] is a Gabor pattern (two-dimensional Gaussian multiplied by a sinusoidal grating) in sine phase with peak spatial frequency of 0.375 cycle/degree and a one-octave spatial bandwidth at half height. The temporal presentation was a Gaussian pulse centered in a 600 ms window with a standard deviation (SD) of 100 ms. These spatial and temporal properties yield a stimulus resistant to variations in retinal illumination and peripheral defocus, [5], [6] with low test–retest variability in glaucomatous defects [4].

### 3.4 Threshold estimation

At the end of each test, threshold at each location was estimated from the mean of the last two reversals. In addition, at each location data were fit with a psychometric function, using maximum likelihood estimation, [2] and the resulting parameters were sensitivity, slope, and false negative rate. The difference (in log units) between the mean of reversals and the sensitivity estimate is listed in the spreadsheet as the “log10Ratio”, which can be used as a reliability estimate [2] but was not used for the analyses in this Excel file.

### 3.5 Reliability criteria

Tests with lid artifacts were removed before reliability criteria were applied. [9]. For the publication, [1] a reliable CSP test was defined as one with false negative rate no greater than 5%, false positive rate no greater than 10% and fixation loss no greater than 30%. As shown in the first column of the first spreadsheet, these criteria removed 4 out of 107 people and 79 of 491 tests.

## 4 Spreadsheet and indices

Four indices were computed: MS, Inner, Outer, PDI.

The “mean sensitivity” (MS) index was computed as the average log contrast sensitivity across all 26 locations tested by CSP.

The “Peripheral Depression Index” (PDI) was computed as the difference between outer and inner log contrast sensitivities. A negative value for PDI means that the outer sensitivity was lower than the inner sensitivity, and a PDI of zero of greater means that outer sensitivity was equal to or greater than inner sensitivity.

**Fig. 1:**
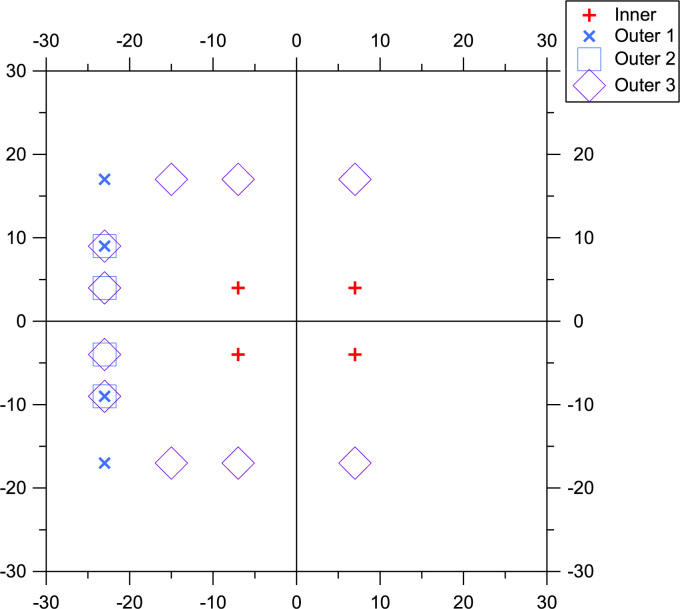
Locations used for the three PDI indices. All three use the same 4 locations to compute inner sensitivity, and different locations for outer sensitivity.

Inner sensitivity was computed as mean log contrast sensitivity for the four inner locations 8° from fixation (Fig. 1, red **+**).

Outer sensitivity was computed as mean log contrast sensitivity for three different sets of outer locations, yielding three different PDI values.

For PDI1, the Outer1 values used 4 locations 25−29° from fixation in the nasal visual field (Fig. 1, blue **X**).

For PDI2, the Outer2 values replaced the 2 locations 29° from fixation with two locations 23° from fixation (Fig. 1, light blue box).

For PDI3, the Outer3 values added 6 more locations to those used in Outer2, at vertical eccentricities of ±17° and horizontal eccentricities from 17° nasal to 7° temporal (Fig. 1, violet diamond).

### 4.1 Excel worksheets

The data are in the Excel file “Data In Brief.xlsx”, which has four worksheets:

The first worksheet. “All 491 tests” has all data from the initial 107 people, before reliability criteria were applied. This is intended to enable other researchers to apply their own reliability criteria. The first column codes reliability for a test as “YES” if all three criteria are passed, and if not lists which criteria: FN (mean false negative rate across all 26 locations), FP (false positive rate from blank trials), FL (fixation loss from stimulus presentations at the blind spot). Only those tests scored “YES” were used to compute indices in the other worksheets.

The second worksheet, “Indices Comparisons”, shows the mean values for each person across all reliable tests: inner, outer, PDI (which equals outer minus inner), MS (mean log contrast sensitivity across all 26 locations), mean age across all reliable tests, and number of reliable tests. This is shown for all three sets of Outer and PDI indices.

The third worksheet, “SDs for 3 indices” shows the standard deviations across tests for those with 7–10 tests, shown for all three sets of Outer and PDI indices.

The fourth worksheet, “CSP Location” shows the *X* and *Y* coordinates for each testing location. Note that locations for left and right eyes are not mirror symmetric.
